# The regulatory roles of the E3 ubiquitin ligase NEDD4 family in DNA damage response

**DOI:** 10.3389/fphys.2022.968927

**Published:** 2022-08-26

**Authors:** Xinxin Lu, Haiqi Xu, Jiaqi Xu, Saien Lu, Shilong You, Xinyue Huang, Naijin Zhang, Lijun Zhang

**Affiliations:** ^1^ Department of Hematology, the First Affiliated Hospital of China Medical University, Shenyang, LN, China; ^2^ Department of Hematology, General Hospital of PLA Northern Theater Command, Shenyang, LN, China; ^3^ Department of Cardiology, the First Affiliated Hospital of China Medical University, Shenyang, LN, China

**Keywords:** E3 ubiquitin ligase, NEDD4 family, DNA damage response (DDR), DNA damage, ubiquitin-proteasome system

## Abstract

E3 ubiquitin ligases, an important part of ubiquitin proteasome system, catalyze the covalent binding of ubiquitin to target substrates, which plays a role in protein ubiquitination and regulates different biological process. DNA damage response (DDR) is induced in response to DNA damage to maintain genome integrity and stability, and this process has crucial significance to a series of cell activities such as differentiation, apoptosis, cell cycle. The NEDD4 family, belonging to HECT E3 ubiquitin ligases, is reported as regulators that participate in the DDR process by recognizing different substrates. In this review, we summarize recent researches on NEDD4 family members in the DDR and discuss the roles of NEDD4 family members in the cascade reactions induced by DNA damage. This review may contribute to the further study of pathophysiology for certain diseases and pharmacology for targeted drugs.

## Introduction

Ubiquitination is a reversible biological process in which proteins are post-translationally modified through a series of multiple catalytic steps, leading to protein degradation by the proteasome or lysosome. A wide range of proteins, including membrane proteins, cell cycle regulators, transcription factors, tumor suppressors and oncogenes, are ubiquitinated (Goel et al., 2015; Zhang et al., 2020a; Zhang et al., 2020b). Therefore, protein ubiquitination is involved in regulating a variety of biological processes (Chen and Matesic, 2007). The ubiquitin-proteasome system is composed of ubiquitin activating enzyme (E1), ubiquitin conjugation enzyme (E2) and ubiquitin ligase (E3). Ubiquitin is activated by E1 and transferred from E2 to substrate mediated by E3 (Xie et al., 2021). E3 ligases can be divided into three classes according to its structure and ubiquitin transfer mechanism: Really interesting new gene (RING)-type E3 ligases, Homologous to E6-AP COOH terminus (HECT)-type E3 ligases and Ring between Ring (RBR)-type E3 ligases (Qie, 2022). RING-type E3 ligases transfer ubiquitin from E2 to substrates directly, while HECT-type E3 ligases and RBR-type E3 ligases transfer ubiquitin in two steps: ubiquitin is transferred from E2 to E3 firstly and from E3 to substrates secondly (Cruz Walma et al., 2022). All seven lysine (K6, K11, K27, K29, K33, K48, and K63) and N-terminal M1 residues form linkage points during chain extension, among which K48 and K63 are mostly studied (Ikeda and Dikic, 2008; Zhang et al., 2020c). K63-linked Ub chains are involved in DDR pathways and NF-κB pathways as previously reported (Bennett and Harper, 2008) and K29/K33-linked Ub chains participate in the regulation of AMP-activated protein kinase (Al-Hakim et al., 2008). Most of E3 ubiquitin ligases belong to the RING family. The HECT-type E3 ligase in humans contains 28 members, and the neural precursor cell–expressed developmentally down-regulated 4 (NEDD4) family is the largest group (Manning and Kumar, 2018; Liu et al., 2021). Multiple studies have demonstrated that HECT E3 ubiquitin ligases are involved in various cellular activities such as protein transport (d'Azzo et al., 2005), subcellular localization (Laine and Ronai, 2007), immune response (Liu, 2004), viral infection (d'Azzo et al., 2005), DNA damage response (DDR) (Harvey and Kumar, 1999; Mohiuddin et al., 2016), oxidative stress (Zhang et al., 2021a) apoptosis (Li et al., 2008a; Kim et al., 2013; Li et al., 2013), cell cycle progression (Goto et al., 2017) and signal transduction (Michnov et al., 2012).

**FIGURE 1:**
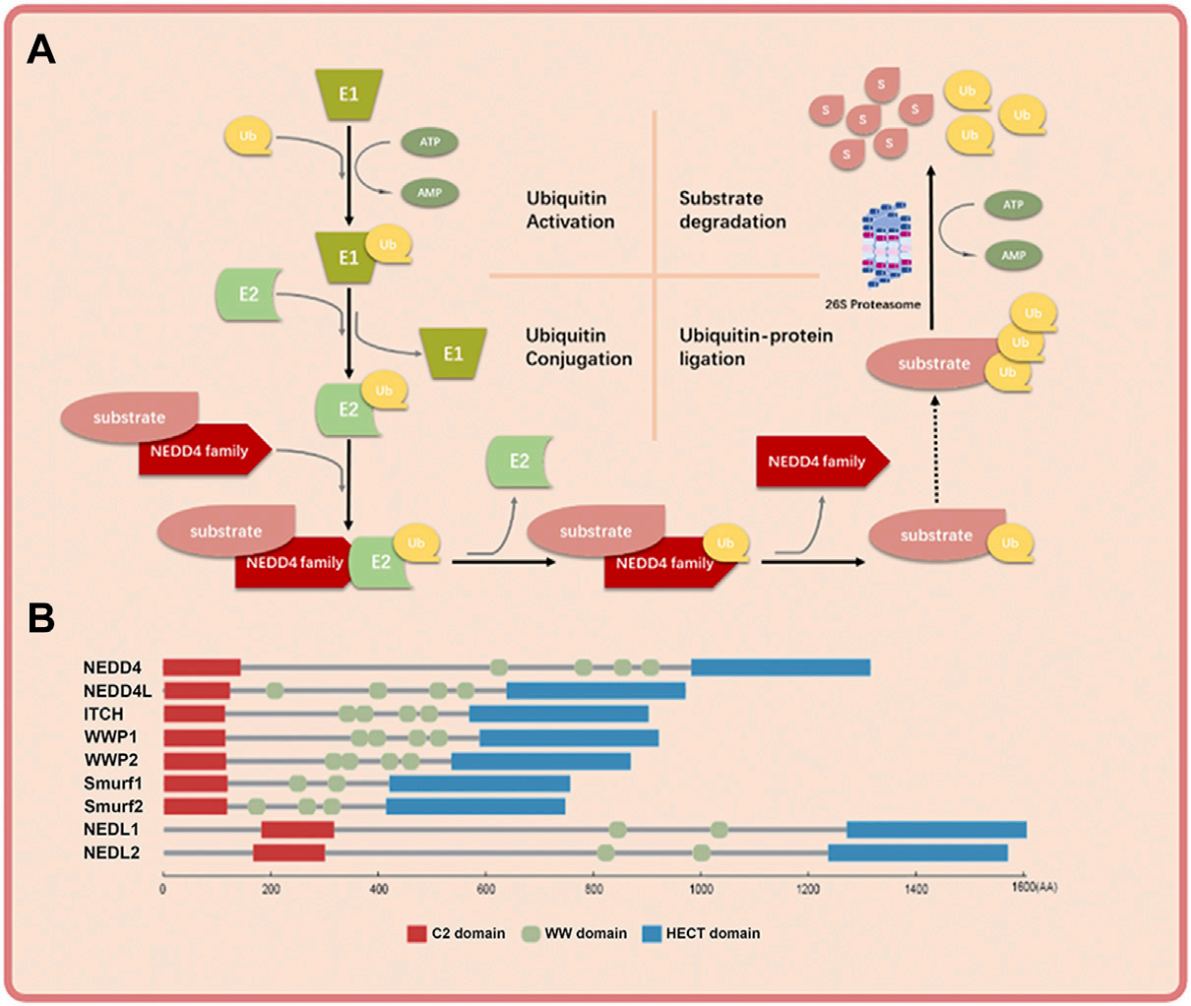
The basic mechanism of NEDD4 family in ubiquitin-proteosome system and the schematic structure of NEDD4 family members. **(A)** Ubiquitin is activated by ubiquitin activating enzyme (E1), transferred by ubiquitin conjugation enzyme (E2) to NEDD4 family (E3), and covalently bound to substrate protein by NEDD4 family. The polyubiquitinated substrate protein is recognized and degraded by 26s proteosome finally. **(B)** The NEDD4 family gene mainly has three functional domains: C2 domain, where the amino terminus can bind to cell membrane, WW domain, which is the central region of protein-protein interaction, and HECT domain, where the carboxyl terminus has ubiquitin-protein connection.

The NEDD4 family consists of nine members: NEDD4 (also known as NEDD4-1), NEDD4L (also known as NEDD4-2), ITCH (also known as AIP4), WWP1 (also known as AIP5), WWP2 (also known as AIP2), SMUEF1, SMURF2, NEDL1 (also known as HECW1) and NEDL2 (also known as HECW2) (Scheffner and Kumar, 2014). They work through binding to both E2-ub thioester and substrate and transferring ubiquitin to substrate finally (Mathieu et al., 2021) (Figure 1A). NEDD4 family proteins have three functional domains: the C2 domain in the amino terminus that binds the cell membrane; the WW domain in the central region that mediates protein-protein interaction; and the HECT domain in the carboxyl terminus that promotes transfer of ubiquitin to substrate proteins (Figure 1B) (Kumar et al., 1997). The C2 domain is a calcium ion–binding domain with a length of about 120 amino acids and binds to phospholipids, inositol, polyphosphates and some proteins (Plant et al., 1997). The WW domain contains 35–40 amino acids and interacts with the PY(PPXY) motif or phosphorylated serine/threonine residues in the substrate protein (Lu et al., 1999). The HECT domain consists of about 350 residues and is responsible for the transfer of ubiquitin to lysine residues in the substrate proteins (Ingham et al., 2004). Because of the diversity of WW domain (Ingham et al., 2004) and substrates (Dodson et al., 2015), the NEDD4 family proteins exert various cellular roles.

**FIGURE 2:**
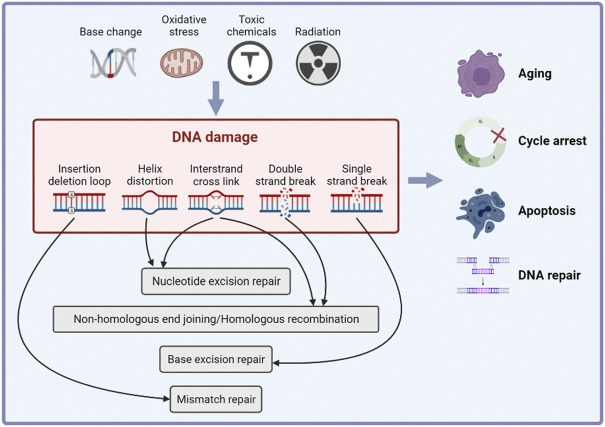
The inducements and effects of DNA damage response. Cells initiate a series of cascade reactions in response of endogenous of exogenous DNA damage inducements, which is known as DNA damage response (DDR). The main effects of DDR include cell cycle arrest, apoptosis, aging and DNA repair.

DNA damage is the alteration of DNA nucleotide sequence caused by endogenous factors (such as base changes or oxidation) or exogenous genotoxic factors (Molinaro et al., 2021). DNA damage is associated with aging (Vijg, 2021), cancer development (Perkhofer et al., 2021; Rivas-Domínguez et al., 2021) and some systemic diseases (Zhang et al., 2021b; Hu et al., 2021; Wang et al., 2021). To repair DNA damage and maintain the integrity of the genome, cells initiate a series of cascade reactions that detect DNA damage and transmit information, collectively known as the DNA damage response (DDR). The main outcomes of the DDR include cell cycle arrest, apoptosis, aging and DNA repair (Figure 2) (Visser and Thomas, 2021). DNA repair mechanisms vary depending on the type of DNA damage. Base excision repair (BER) repairs DNA single strand damage. Mismatch repair (MMR) detects and removes the insertion or deletion of mismatched nucleotides during DNA replication. Nucleotide excision repair (NER) repairs large or helically destabilizing DNA lesions. DNA double-strand breaks are repaired by homologous recombination (HR) and non-homologous end joining (NHEJ) (Rivas-Domínguez et al., 2021). It is reported that Ataxia-telangiectasia mutated (ATM), ATM- and RAD3-related (ATR) and DNA-dependent protein kinase (DNA-PK) play important roles in DDR (Blackford and Jackson, 2017). The ATM kinase phosphorylates proteins in response to recognition of DNA double strand breaks (DSBs) and ATR stops the DNA replication process and promotes DNA repair. DNA-PK mainly participates in NHEJ and activates a small subset of proteins (Kiss et al., 2021). The point of DDR is repairing DNA damage and activating cell death if the damage is irreparable, and thus preventing the transmission of potentially harmful DNA mutations (Goff et al., 2021).

In recent years, several studies have shown that members of the NEDD4 family participate in and regulate multiple DDR pathways to maintain genome integrity. Therefore, this review aims to summarize the mechanistic role of NEDD4 family members in DDR and provide a general understanding of the roles of NEDD4 family members in the cascade reactions induced by DNA damage. This review may also contribute to the further study of pathophysiology for certain diseases and pharmacology for targeted drugs.

## NEDD4

### NEDD4 function

NEDD4, the first identified member of the NEDD4 family, was first isolated from neural progenitor cells in 1992 as a factor with mRNA levels down-regulated during mouse brain development (Kumar et al., 1992; Yang and Kumar, 2010). The human NEDD4 gene, located on chromosome 15Q21.3, contains 33 exons and encodes a protein with a molecular weight of 120 kDa. NEDD4 is expressed in heart, lung, brain, kidney, and other tissues (Fagerberg et al., 2014) and mainly localizes in the cytoplasm and specifically around the nucleus (Anan et al., 1998; Putz et al., 2008). NEDD4 was originally shown to regulate water and electrolyte balance by controlling the abundance of sodium channels in epithelial cells. Subsequent studies demonstrated that NEDD4 regulates embryonic and tumor development as an E3 ligase (Huang et al., 2019). In yeast and mammalian cells, NEDD4 regulates intracellular sorting and transport (Yalcin et al., 2019; Xie et al., 2020). NEDD4 also functions in protein degradation through the polyubiquitination of K48 and K63 sites (Xu et al., 2015; French et al., 2017; Sluimer and Distel, 2018) or monoubiquitin of K6 and K27 sites (Murillas et al., 2002; Fukushima et al., 2015).

### NEDD4 and DDR

Mdm2 is an E3 ubiquitin ligase and important negative regulator of tumor suppressor protein p53 (Fang et al., 2000). Mdm2 overexpression leads to the inactivation of p53 (Lai et al., 2001). NEDD4 regulates the stability of Mdm2 in cells, thus contributing to regulation of p53 in the DNA damage response. NEDD4 physically interacts with Mdm2 via the RING domain of Mdm2 and promotes Mdm2 ubiquitination. In NEDD4 knockout cells, the expression level of p53, Mdm2-dependent behavior and DDR were increased. Therefore, NEDD4 is an important component of p53 pathway that influences Mdm2 stabilization in the DNA damage response (Xu et al., 2015).

**FIGURE 3:**
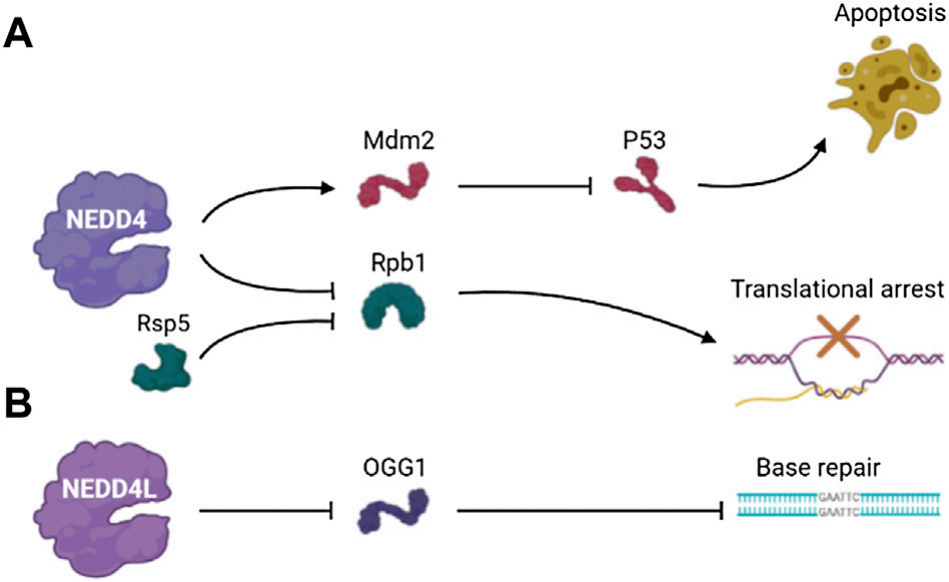
The pathways involved in DDR of **(A)** NEDD4 and **(B)** NEDD4L.

RNA polymerase II (Rpb1) is the largest subunit of RNA polymerase II (Pol II), a substrate of Rsp5 that is an E3 ubiquitin-protein ligase and is essential for the synthesis of mRNA and transcript synthesis following repair (Somesh et al., 2005; Calvo, 2020). The ubiquitination and degradation of Rpb1 participates in the transcriptional arrest induced by DNA damage. The ubiquitination of Rpb1 mediated by Rsp5 is part of the DNA damage response, and Rpb1 is specifically identified to enhance DNA damage response. Beaudenon et al. demonstrated that NEDD4 mediated UV-induced degradation of Pol II by ubiquitination of Rpb1. This finding suggests that an irreversible disassembly of transcription complexes by the degradation of the major catalytic subunit of Pol II works in response to DNA damage (Beaudenon et al., 1999) (Figure 3A).

### NEDD4L

#### NEDD4L function

NEDD4L is the closest homologue of NEDD4 in the NEDD4 family (Harvey and Kumar, 1999; Goel et al., 2015). The human NEDD4L gene, located on chromosome 18Q21.31, contains 38 exons and can produce multiple spliced mRNAs (Chen et al., 2001; Harvey et al., 2001). NEDD4L transcripts exist in many tissues, especially in liver, kidney, heart and lung (Dunn et al., 2002; Araki et al., 2008), and NEDD4L is down-regulated in melanoma (Cui et al., 2020), colorectal cancer (Yang et al., 2020), lung cancer (Wang et al., 2019) and other cancers, indicating NEDD4L is a tumor suppressor. NEDD4L regulates many membrane proteins, such as epithelial and voltage-gated sodium channels (Goel et al., 2015; Gao et al., 2021). It mediates the Wnt/β-catenin and TGF-β signaling pathways and plays a key role in preventing the progression of systemic chronic kidney disease (Manning et al., 2021). NEDD4L catalyzes K29-linked ubiquitination of cysteine residues in TRAF3, which is of vital importance against innate immunity to viruses (Gao et al., 2021). In addition, NEDD4L is also involved in virus budding by ubiquitination and activation of ESCRT-I (Chung et al., 2008).

#### NEDD4L and DDR

Eight-oxyguanine lesions are premutagenic lesions that lead to GC to TA translocation, and 8-oxyguanine DNA glycosylase (OGG1) is a major DNA glycosylase that removes 8-oxyguanine lesions from DNA (Boiteux and Radicella, 2000). OGG1 plays a regulatory role in controlling gene expression by the removal of 8-oxyguanine lesions in guanine-rich promoter sequences, leading to transcriptional activation of guanine quadruplet structures (Fleming et al., 2017; Wang et al., 2018). Hughes and Parsons showed that ubiquitination of OGG1 on lysine 341 by NEDD4L inhibited its DNA glycosylase/lyase activity, and the dysregulation of OGG1 increased DNA damage (Hughes and Parsons, 2020). This result suggests that the regulation of OGG1 protein level plays an important role in the repairing of bases and the maintenance of genomic stability, and this process involves the ubiquitination and degradation mediated by the NEDD4L E3 ubiquitin ligase (Figure 3B).

### ITCH

#### ITCH function

ITCH was originally identified by genetic studies of mouse fur color changes and its deletion leads to the itchy phenotype of persistent skin scratching and multi-organ inflammation (WL et al., 1998). The human ITCH gene is located on chromosome 20q11.22 and encodes 864 amino acids (Infante et al., 2019; Li et al., 2020). It contains the typical domains of the NEDD4 family and four WW domains that interact with substrates. ITCH regulates a series of signaling pathways by promoting polyubiquitination of over 50 target proteins, including C-Jun, C-flip, LATS1, P63, P73, TCR-ζ and BRAF. ITCH not only catalyzes K48 polyubiquitination for proteolytic hydrolysis, but it also promotes the polyubiquitin chains linked to K63, K27 and K33 (Yin et al., 2020).

#### ITCH and DDR

P73, a member of the p53 family of transcription factors, is up-regulated in response to DNA damage and induces cell cycle arrest and apoptosis. In 2005, Rossi found that ITCH bound to and ubiquitinated p73, leading to rapid proteasome-dependent degradation of p73 (Rossi et al., 2005). Studies have shown that YES-associated protein (Yap1) binds to p73 and enables p73 to escape ITCH-mediated ubiquitination by competing with ITCH (Levy et al., 2007). Under normal conditions, Yap1 enhances transcriptional activation of ITCH through Runx binding sites and causes degradation of p73. Yap1 is phosphorylated by c-abl at Tyr357 in response to DNA damage and failed to activate Runx and promote ITCH transcription (Levy et al., 2008a). The resulting decreased ITCH level leads to accumulation of p73 (Levy et al., 2008b). Hansen demonstrated that the ubiquitination of p73 mediated by ITCH played an important role in regulating DNA damage–induced apoptosis. ITCH knockout increased apoptosis in response to DNA damage agents used in chemotherapy, and reintroduction of ITCH into fibroblasts from ITCH-deficient mice reduced cell death after DNA damage (Hansen et al., 2007). These results indicated that inhibition of ITCH activity was important to modulate the chemosensitivity of DNA damage agents and targeting ITCH may help potentiate the effect of chemotherapeutic drugs on cancers.

ATM, which is induced by DNA damage, regulates cellular responses mainly through the phosphorylation of downstream target proteins (Shiloh, 2003; Bhatti et al., 2011). Santini et al. found that the S161 residue in ITCH was necessary for ATM-dependent ITCH activation. ATM enhanced ITCH enzyme activity and promoted the ubiquitination and degradation of c-FLIP-L (Gao et al., 2004; Chang et al., 2006), which participates in DDR and the modulation of death receptor signaling (Santini et al., 2014).

WW domain oxidoreductase (WWOX) is a tumor suppressor that spans the common fragile site FRA16D on chromosomes and plays an important role in DDR (Bednarek et al., 2000). WWOX deletion results in impaired DNA repair. WWOX is ubiquitinated at lysine 274 by ITCH and interacts with ATM when DNA single strand breaks occurs, thereby regulating ATR checkpoint and leading to cell cycle arrest to repair damaged DNA (Abu-Odeh et al., 2014; Abu-Odeh et al., 2015). These data indicated that regulation of WWOX by ITCH plays an important role in genome stability and clonal expansion of neoplastic cells.

**FIGURE 4:**
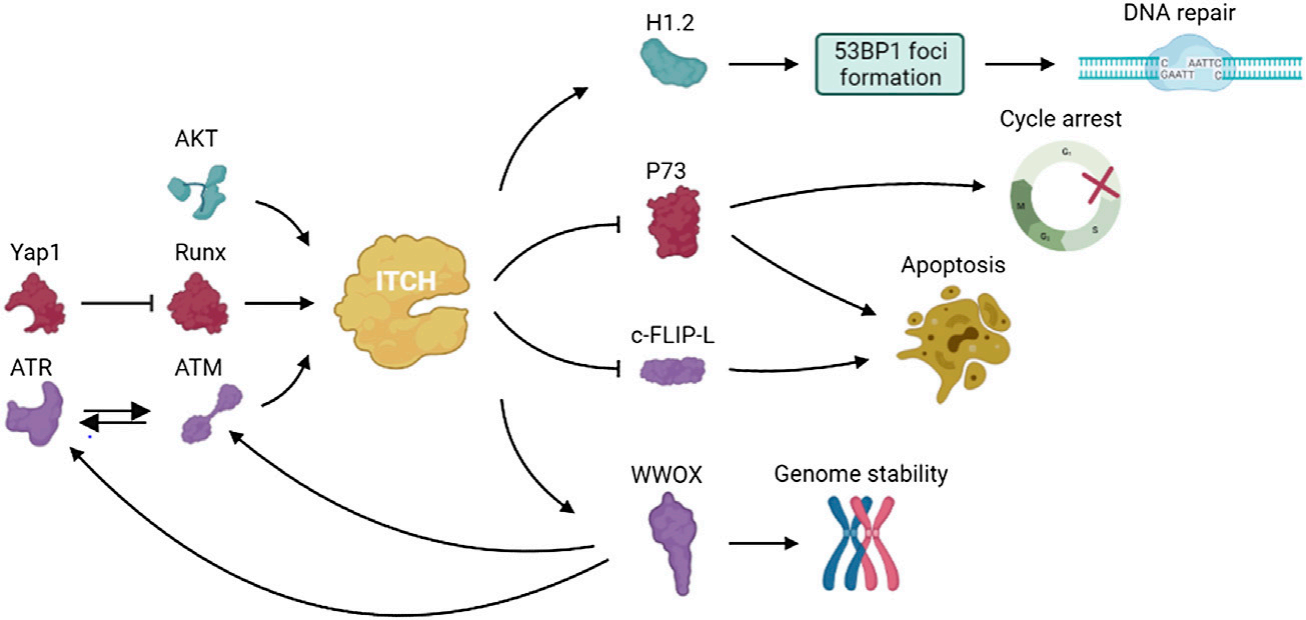
The pathways involved in DDR of ITCH.

Down-regulation of the DDR can enable the uncontrolled proliferation of invasive tumors. Studies showed that ITCH is highly expressed in triple negative breast cancer (TNBC) and modulated DDR in TNBC (Chang et al., 2019). Phosphorylation of ITCH at Ser257 by AKT leads to the nuclear localization of ITCH and the ubiquitination of H1.2. ITCH mediates H1.2 polyubiquitination and inhibits the formation of 53BP1 foci, which play a part in cell cycle checkpoints and DNA damage repair (Thorslund et al., 2015). These results demonstrated that the AKT-activated ITCH-H1.2 axis may lead to DDR inhibition in TNBC cells, which can offset replication stress and improve the survival and growth potential of tumor cells (Figure 4).

### WWP1

#### WWP1 function

The gene encoding human E3 ubiquitin ligase WWP1 is located at 8q21 (Chen et al., 2007). WWP1 is a 922 amino acid protein that contains one C2 domain, four WW domains and one HECT domain (Zhi and Chen, 2012). The C2 domain determines the subcellular localization of the molecule, while the WW domain binds to the proline-rich sequence (PY motif) of the substrate protein (Hoshino et al., 2020). Studies have shown that WWP1 regulates a variety of cellular biological processes such as protein transport and degradation, signal transduction and transcription (Jia et al., 2021).

#### WWP1 and DDR

**FIGURE 5:**
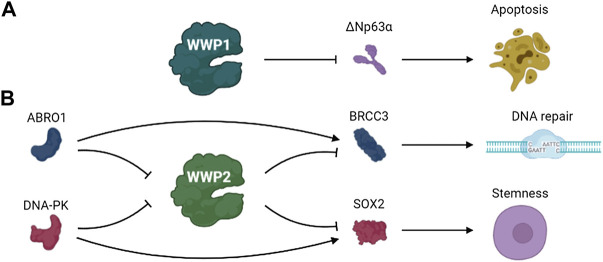
The pathways involved in DDR of **(A)** WWP1 and **(B)** WWP2.

ΔNp63α, an isomer of the protein encoded by p63 gene, is homologous to p53 and plays a role in cell survival and proliferation (Mills et al., 1999; Kurinna et al., 2021; Xu et al., 2021). Previous studies identified ΔNp63α as a ubiquitination substrate of WWP1 (Li and Xiao, 2014). DNA damage stimulates WWP1 transcription, and WWP1 knockout eliminated the down-regulation of ΔNp63α expression induced by DNA damage and salvaged the apoptosis caused by DNA damage (Chen et al., 2017). The expression of WWP1 is up-regulated under the stimulation of DNA damage chemotherapeutic drugs. These results indicate that WWP1 may be involved in the effects of chemotherapeutic drugs (Li et al., 2008b) (Figure 5A).

### WWP2

#### WWP2 function

In 1997, Wood and others identified WWP2 as binding to atrophin-1 by yeast two-hybrid and vitro binding analysis and named WWP2 as atrophin-1 interacting protein 2 (AIP2) (Zhang et al., 2019). The same gene locus produces three protein subtypes: full-length WWP2 (WWP2-FL, 870 AA), N-terminal WWP2-N (WWP2-N, 336 AA), and C-terminal WWP2-C (WWP2-C, 440 AA) (Soond and Chantry, 2011; Chen et al., 2014). WWP2 is widely expressed in the heart, placenta, lungs, liver, muscles, kidneys, pancreas and brain (Xu et al., 2009). Several substrates of WWP2 have been identified such as Oct4 (Liao and Jin, 2010), PTEN (Fang et al., 2020), TIRF (Yang et al., 2013), Septin4 (Zhang et al., 2020b) and PARP1 (Zhang et al., 2020a). WWP2 regulates various cellular physiological processes by regulating ubiquitin-dependent degradation of these proteins (Shao et al., 2016; Mokuda et al., 2019; Zhang et al., 2019). Identification of new substrates for WWP2 will expand the understanding of its physiological functions (Zhang et al., 2021c).

#### WWP2 and DDR

BRCA1/BRCA2-containing complex subunit 3 (BRCC3) is a lysine 63–specific deubiquitinating enzyme that is involved in various biological processes such as DNA damage repair (Ng et al., 2016; Rabl et al., 2019). WWP2 regulates the ubiquitination and degradation of BRCC3, thus regulating the functions of BRCC3. ABRO1, a subunit of the BRCC36 isopeptidase complex (BRISC), prevents WWP2-mediated BRCC3 ubiquitination and enhances BRCC3 stability by competing with WWP2 to bind to BRCC3. Thus, the stability of BRCC3 regulated by WWP2 and ABRO1 may play a role in DDR (Zhang et al., 2021c).

Glioblastoma, a lethal primary brain tumor, contains glioma stem cells that promote malignant progression and therapeutic resistance. SOX2 plays crucial roles in maintaining the self-renewal potential of normal stem cells in glioblastoma (Masui et al., 2007; Kim et al., 2008; Boumahdi et al., 2014; Zhang et al., 2020d). S251 of SOX2 is phosphorylated by DNA-PK under normal conditions, preventing the ubiquitination and degradation of SOX2 mediated by WWP2 and maintaining the stem status of glioma stem cells. The separation of DNA-PK from SOX2 because of DNA double-strand breaks promoted WWP2 binding with SOX2 and failed to stabilize SOX by preventing the ubiquitination and degradation of SOX2 mediated by WWP2, thus promoting the differentiation of glioma stem cells (Fang et al., 2021). These data indicate that DNA damage triggers glioma stem cell differentiation through precise regulation of SOX2 stability by DNA-PK and WWP2, suggesting this pathway may be a possible therapeutic target of glioblastoma (Figure 5B).

### Smurf1

#### Smurf1 function

The human SMURF1 gene is located on chromosome 7q22.1 (Xia et al., 2021). Smurf1 is usually found in the cytoplasm, but sometimes localizes in the plasma membrane or nucleus by interacting with other proteins (Suzuki et al., 2002; Horiki et al., 2004). Smurf1 contains one C2 domain, two WW domains (WW1 and WW2) and one HECT domain. Smurf1 substrates are ubiquitinated either through direct interactions between the PY motifs in substrates and the WW domain or through indirect interactions mediated by adaptor proteins (Cao and Zhang, 2013). Nearly 40 substrate proteins of Smurf1 have been discovered. Smurf1 participates in many processes including tumorigenesis, embryogenesis, regulation of cell localization and maintenance of homeostasis through the ubiquitin proteasome pathway by targeting substrate proteins (Shimazu et al., 2016; Feng et al., 2019; Scott et al., 2020).

#### Smurf1 and DDR

**FIGURE 6:**
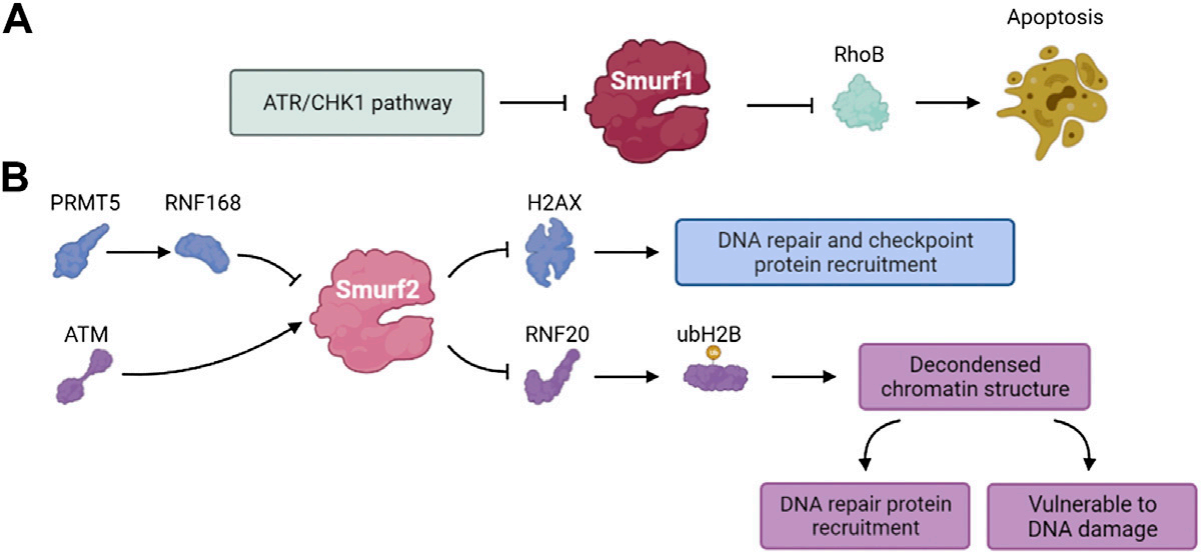
The pathways involved in DDR of **(A)** Smurf1 and **(B)** Smurf2.

The Rho GTPase family of proteins are key regulators of actin cytoskeletal dynamics and participate in regulating the cell cycle, gene expression, vesicle transport and cell polarity (Kaibuchi et al., 1999; Bustelo et al., 2007). RhoB is a member of the Rho GTPase family (Gutierrez et al., 2019). Some studies have shown that RhoB deficiency significantly reduced the apoptosis of transformed cells induced by DNA damage (Liu et al., 2001). The ATR/Chk1 signaling pathway, activated by DNA damage, mediates the phosphorylation and self-degradation of Smurf1, leading to the accumulation of RhoB and promoting cell apoptosis (Wang et al., 2014). These results suggest that Smurf1 is involved in the DNA damage response and regulates various cell functions (Figure 6A).

### Smurf2

#### Smurf2 function

The human Smurf2 gene is located on chromosome 17. Similar to other members of the NEDD4 family, Smurf2 contains one C2 domain, three WW domains with two conserved tryptophan residues each and one HECT domain (Bai and Ying, 2020). The WW domain is responsible for substrate recognition through specific binding to the PPXY motif (Lin et al., 2000). Smurf2 was initially found to negatively regulate TGF-β/BMP signaling by ubiquitination and degradation of substrates, preventing overactivation of TGF-β/BMP signaling (Kushioka et al., 2020). Smurf2 also contributes to genomic stability, cell polarity, tissue homeostasis, embryogenesis and tumorigenesis (Koganti et al., 2018; Bai and Ying, 2020; Shepley-McTaggart et al., 2021).

#### Smurf2 and DDR

Phosphorylation of histone H2AX (γH2AX) and localization of γH2AX to DSB sites play a vital role in the DDR. The γH2AX foci formation functions as recruiting other DDR effectors and is a mark of early step of DDR (Celeste et al., 2003; Yuan et al., 2010; Machitani et al., 2020). Du et al. found that the PRMT5-RNF168-Smurf2 cascade regulated the protein stability of H2AX and confirmed Smurf2 was essential to DDR. RNF168 and SMURF2 regulate the expression of H2AX as stabilizers and destabilizers of H2AX, respectively. Inhibition of PRMT5 attenuates the expression of RNF168 and causes Smurf2 to destabilize H2AX by ubiquitination and degradation. Thus, Smurf2 regulates the levels of H2AX in the PRMT5- RNF168-SmurF2 cascade and the related effect in the process of DDR (Du et al., 2019).

Genomic ablation of Smurf2 results in dysregulation of the DNA damage response and genomic stability. Smurf2 is phosphorylated by ATM at S384 when DNA damage occurs, and its phosphorylation is required for its interaction with ring finger protein 20 (RNF20) (Blank et al., 2012). Smurf2-mediated ubiquitination and degradation of RNF20 contributes to a negative feedback loop that regulates DSB repair. The mono-ubiquitination of H2B mediated by RNF20 relaxes chromatin, which plays dual roles in DDR. The SMURF2-mediated RNF20 ubiquitination leads to down-regulation of H2B, and the chromatin compaction protects cells from DNA damage but disturbs the recruitment of DNA repair proteins (Tang et al., 2020). Ayyathan et al. also demonstrated that the inactivation of Smurf2 triggered a variety of changes in cellular activities including cell mobility, self-replication and DNA damage repair (Manikoth Ayyathan et al., 2020). Thus, Smurf2 is a key regulator of the DDR, chromatin structure regulation and genome integrity maintenance (Figure 6B).

## Conclusion and prospects

The DDR functions to maintain the integrity of the genome is necessary for the growth and survival of cells and organisms. Activating DDR signaling pathways promote cellular homeostasis and survival in health and disease (Chatzidoukaki et al., 2020). While, an impaired DDR is related to some disease such as autosomal dominant polycystic kidney disease and Alzheimer’s disease (Farmer et al., 2020; Zhang et al., 2021b). An abnormal DDR process leads to a change of cellular activities facing to stimulus, which provides a clue for treating some diseases. In this review, we summarize the recent literature on the function and regulation of NEDD4 family members in DDR (Table 1). It showed NEDD4 family play important roles in maintaining genome integrity and stability by regulating different substrate proteins and participating in various pathways involved in DDR. Therefore, identification of new target proteins of the NEDD4 family may help elucidate more mechanisms of DDR, which may provide an idea to the study of cellular homeostasis and the balance between health and disease, and contribute to the further study of pathophysiology for certain diseases and pharmacology for targeted drugs.

**TABLE 1:** The summary of E3 ubiquitin ligase NEDD4 family regulatory roles in DDR.

Enzymes	Mechanisms	References
NEDD4	NEDD4 regulates the stability of Mdm2 and contribute to the ubiquitination and degradation of P53 by Mdm2 in DNA damage response	Xu et al. (2015)
	NEDD4 ubiquitinates and degrades Rpb1 under UV-induced DNA damage	Beaudenon et al. (1999)
NEDD4L	NEDD4L stimulates ubiquitylation of OGG1 particularly on lysine 341, inhibiting DNA glycosylase/lyase activity, increases the formation of lethal intermediate DNA lesions and decreases DNA repair capacity	Hughes and Parsons (2020)
ITCH	In response to DNA damage, phosphorylated Yap1 doesn’t co-active Runx in supporting Itch transcription and sharply reduced Itch levels alleviate the ubiquitination of P73, contributing to the accumulation and activation of P73	(Levy et al., 2007; Levy et al., 2008a; Levy et al., 2008b)
	Inhibition of the activity of Itch and subsequent accumulation of p73 are very important in regulating sensitivity to DNA damaging agents used in chemotherapy	Hansen et al. (2007)
	ATM activity enhances ITCH enzymatic activity, which in turn drives the ubiquitination and degradation of c-FLIP-L	(Gao et al., 2004; Chang et al., 2006; Santini et al., 2014)
	ITCH ubiquitination modifies WWOX lys274 to accumulate and interact with ATM during DNA single strand breaks, thereby regulating ATR checkpoint and leading to cell cycle arrest to repair damaged DNA.	(Abu-Odeh et al., 2014; Abu-Odeh et al., 2015)
	Phosphorylation of ITCH by AKT at Ser257 leads to the nuclear localization of ITCH and ITCH mediated H1.2 polyubiquitination inhibits the formation of 53BP1 foci, which play an important role in cell cycle checkpoints and DNA damage repair	(Thorslund et al., 2015; Chang et al., 2019)
WWP1	DNA damage could stimulate WWP1 transcription and WWP1 knockout can eliminate the down-regulation of ΔNp63α expression, which could be ubiquitinated and degraded by WWP1, induced by DNA damage, and salvage the apoptosis caused by DNA damage	(Li and Xiao, 2014; Chen et al., 2017)
	The expression of WWP1 is up-regulated under the stimulation of DNA damage chemotherapeutic drugs	Li et al. (2008b)
WWP2	WWP2 regulates the ubiquitination and degradation of BRCC3, thus regulating the related functions of BRCC3 such as DNA repair and immune responses. While ABOR1 competes with WWP2 in binding to BRCC3	Zhang et al. (2021c)
	The separation of DNA-PK from SOX2 due to the occurrence of DNA double-strand break promotes the ubiquitination of WWP2 to SOX2, thus promoting the differentiation of glioma stem cells. This pathway may be a possible therapeutic target of glioblastoma	Fang et al. (2021)
Smurf1	the ATR/Chk1 signaling pathway of DNA damage response mediates the phosphorylation of Smurf1 and promotes its self-degradation, leading to RhoB accumulation and promoting cell apoptosis	(Liu et al., 2001; Wang et al., 2014)
Smurf2	Inhibition of PRMT5 attenuates the expression of RNF168 and causes the E3 ubiquitin ligase Smurf2 to destabilize H2AX by poly-ubiquitination, which is essential to DDR.	Du et al. (2019)
	Smurf2-mediated ubiquitination and degradation of RNF20 are controlled by ATM-induced phosphorylation at S384, forming a negative feedback loop regulating DSB repair. Subsequent down-regulation of H2B and chromatin compaction protects cells from DNA damage but disturbs the recruitment of DNA repair proteins	(Blank et al., 2012; Manikoth Ayyathan et al., 2020; Tang et al., 2020)
NEDL1	None	None
NEDL2	None	None
